# Immunomodulation of nutritional formula containing epigallocatechin-3-gallate, ginseng extract, and polydextrose on inflammation and macrophage polarization

**DOI:** 10.3389/fnut.2024.1370608

**Published:** 2024-02-20

**Authors:** Yi Wang, Yaozhong Hu, Zhenhua Niu, Xuejiao Zhang, Dancai Fan, Xuemeng Ji, Huan Lv, Shuo Wang, Yanrong Zhao

**Affiliations:** ^1^Tianjin Key Laboratory of Food Science and Health, School of Medicine, Nankai University, Tianjin, China; ^2^Shanghai M-Action Health Technology Co., Ltd., Shanghai, China

**Keywords:** EGCG, ginseng extract, polydextrose, nutrient complex, macrophage

## Abstract

Single nutrient likes polyphenol or dietary fiber have been exhaustively investigated to validate their positive intervention in health or disease. Meanwhile, the common interaction of inner systems with the nutrient complex has not been well elucidated, which raises the scientific issue of the modulatory effect of the nutrient complex on immunity. The representative prebiotics of epigallocatechin-3-gallate (EGCG), ginseng extract, and polydextrose (PDX) were selected on behalf of the classification of polyphenol, flavone or polysaccharides, and dietary fiber to generally cover the daily food intake in this study to explore their intervention in inflammation and macrophage polarization. The intervention of selected nutrients on inflammation and macrophage polarization has been evaluated against macrophages to unveil their comprehensive effects. The synergistic effect of selected nutrients was demonstrated by inhibiting M1 macrophage polarization and the promotion of M2 macrophage polarization. Then, the nutrient formula was set up to verify the intervention effect, and the results revealed the significant inhibition of cell inflammation and the effect on cell proliferation through promoting the cell cycle in the G2 phase. The nutrient complex could inhibit M1 macrophage polarization to inhibit M1-mediated inflammation and promote M2 macrophages for anti-inflammatory effect and enhance cell phagocytosis. Moreover, the varied intervention effects of the nutrient complex with different formulas could be summarized. In general, the formula containing EGCG, ginseng extract, and PDX was demonstrated to possess an enhanced immunomodulatory effect on cell inflammation and macrophage polarization, which could potentially inspire the investigation of complex nutrients in health and diseases.

## 1 Introduction

It has been widely accepted that varied nutrients are required for daily exercise to benefit the health and function of inner systems, including the immune system. Numerous studies have explored the intervention effect of individual nutrients on health or disease to report their positive effects (1–3). However, the common interaction of the inner systems with the nutrient complex has not been well investigated, nor have its comprehensive effects on health or diseases, hence emphasizing the need for investigation. Thus, the scientific issue concerning the effect of the nutrient complex on macrophage phenotypes was raised to explore the enhanced immune function in this study. The distinct dietary patterns from the west to the east comprise varied nutrients, which increases the difficulty of research proposals to include different types of nutrients as exhaustively as possible. Meanwhile, the widely applied food types all over the world can be classified as representative health-beneficial nutrients or prebiotics such as polyphenols, flavones, and dietary fibers, which address the functionality of the representative food nutrients of tea polyphenols, ginseng, and polydextrose (PDX) that are consumed worldwide. Thus, the representative prebiotics of epigallocatechin-3-gallate (EGCG), ginseng extract, and PDX were selected on behalf of the classification of polyphenol, flavone or polysaccharides, and dietary fiber to generally cover the daily food intake worldwide. More importantly, the intervention effects of the selected nutrients have been stated with the identical effects of anti-oxidative stress, anti-inflammation, and immunity enhancement, etc.; in particular, a large number of studies have shown that these compounds have excellent functions in immune regulation, such as reducing the release of inflammatory factors from macrophages and potentially enlightening the enhanced intervention of their complex (4–6).

The novelty of tea polyphenols relies on the frequent daily intake around the world to elicit intervention on homeostasis and human health. As the most abundant polyphenol in green tea, EGCG has been thoroughly evaluated to identify its effect on inner homeostasis or the progression of various diseases. Previous reports revealed the alleviating effect of EGCG on inflammation-related chronic disease through regulating the process of lipid metabolism (7, 8). Other research unveiled the relieving effect of EGCG on colonic inflammation in a colitis mouse model (9). Relevant studies provided evidence concerning the autoxidation of EGCG and the mediated production of reactive oxygen species (10). The intervention effect of EGCG on macrophages has been reported in a study about acute lung injury, and the results indicated the inhibition of inflammatory macrophage phenotypes (11). Furthermore, the alteration of macrophage polarization from M1 to M2 phenotype was verified in non-alcoholic fatty liver disease (NAFLD) mice (12). Thus, the immune modulatory effect of EGCG has been raised in previous studies.

For selection of flavone resources, ginseng and its extract have been highlighted and focused on to analyze their dominant contents and beneficial effects. The wide application of ginseng as a functional food ingredient or traditional herbal medicine has addressed its beneficial effects on health through regulating inflammation, immunity, and oxidation, etc. To date, it has been developed as frequently ingested prebiotics in food to maintain the health of the inner systems. The published data have demonstrated the main actives of ginseng as ginseng flavone, ginsenosides, and ginseng polysaccharides. Their bio-activities have been widely investigated to report the inhibitory effect on varied diseases such as tumor and inflammation (13–15). The inhibition of ginsenoside on tumor angiogenesis and growth was concluded in an experiment on nude mice (16). Huang et al. reported the immune enhancement of ginseng polysaccharides through sensitizing the tumor immune therapy against the immune checkpoint (17). Yang et al. (18) have studied the intervention effect of ginseng extract on inflammation and oxidative stress in RAW264.7 cells and in a dextran sulfate sodium (DSS)-induced colitis mouse model, revealing that ginseng extract can exert both anti-inflammatory and anti-oxidative functions by targeting the MAPK/NF-κB and p62-Nrf2-Keap1 pathways, as well as autophagy, *in vitro* and *vivo*.

The alternative nutrient recommended for daily intake is dietary fiber, which has been verified to have a positive effect on gastrointestinal homeostasis. As a kind of soluble dietary fiber containing randomly bonded glucose polymer, PDX is thought to be a food prebiotic with impact on health and disease after digestion and fermentation by intestinal microbiota (19). Several cohort studies indicated the alleviating effect of PDX, from fecal bulk to soft stools, as the result of raising defecation frequency (20, 21). Luoto et al. (22) demonstrated the significant inhibitory effect of PDX on viral infection in the respiratory tract of infants. A previous study also revealed that PDX can modulate the gut microbiota and attenuate serum triglyceride and cholesterol levels in mice fed with a western diet for 14 days (23). Hu et al. (24) reported that PDX alleviated serum lipopolysaccharide (LPS) levels and macrophage infiltration in epididymal adipose tissue and resulted in macrophage polarization toward the M2 phenotype, as the result of preventing and treating obesity in high-fat-diet-fed mice, specifically in alleviating glucolipid metabolism disorders and adipose tissue inflammation.

Host immunity consists of two types of defensive strategies: innate and adaptive immunity. As the most studied and focused cells, macrophages are classified as monocytes generally originated from progenitors in bone marrows and identified as innate immune cells with high homogeneity and plasticity (25, 26). Macrophages are classified as different formats in organs and tissue and as recruited or resident cells to be involved the general immune defense or tissue reoccurrence. Macrophage polarization is considered a vital process to determine the functionality of macrophages under different conditions, with the classically activated M1 phenotypes and alternatively activated M2 macrophages. M1 and M2 macrophages play different roles in different processes through regulating different signaling pathways to finally show distinct or even opposite functions. Under a normal state, M1 macrophages are mainly responsible for the phagocytotic capacity to eliminate invaded pathogens or bacteria (27–29). Meanwhile, the inflammatory response is generally understood to potentially aggravate inflammatory diseases. M2 macrophages are believed to be evolved in parasite elimination, tissue remodeling, and allergic diseases (30). Thus, the modulation of macrophage polarization can produce varied biological effects in different microenvironments.

Herein, the nutrient formula, including the prebiotics of EGCG, ginseng extract, and PDX, was prescribed to simulate the daily intake of nutrients to reveal the intervention effect on macrophage polarization. The results demonstrated the positive intervention of the formula on inflammation by inhibiting the release of pro-inflammatory cytokines. More importantly, the immunomodulatory effect on macrophage polarization was validated.

## 2 Materials and methods

### 2.1 Materials

Ginseng extract (Ginsenosides ≥80%, designated as R) and EGCG (designated as E) with purity higher that 94% were purchased from Shanghai Novanat Co., Ltd. (Shanghai, China). PDX (designated as D) with purity higher that 90% was purchased from Tate & Lyle Trading Co., Ltd. (Shanghai, China). The complex of selected nutrients was proposed based on the experimental design and designated as the combination of the abbreviation of the corresponding nutrient (ERD). Lipopolysaccharide (LPS) was purchased from Santa Cruz Biotechnology (California, United States). Cell Counting Kit-8 (CCK-8) reagent was purchased from Meilunbio (Dalian, China). Anti-mouse CD16/32, APC-conjugated anti-mouse F4/80, FITC-conjugated anti-mouse CD86, and PE-conjugated anti-mouse CD206 antibodies were purchased from Thermo Fisher Scientific (Waltham, MA, United States) for flow cytometry analysis. Recombinant mouse IFN-γ and IL-4 were provided by Thermo Fisher Scientific. Enzyme-linked immunosorbent assay (ELISA) kits for TNF-α and IL-1β analysis were purchased from Nanjing Jiancheng Bioengineering Institute (Nanjing, China). Neutral red was from sourced from Aladdin (Shanghai, China). An apoptosis kit was purchased from Thermo Fisher Scientific. All reagents used in this study were of analytical grade unless otherwise indicated.

### 2.2 Cell culture and treatment

The murine macrophage cell line RAW264.7 was from a laboratory collection and was cultured in a DMEM medium (Gibco, Grand Island, NY, United States) with a supplementation of 10% fetal bovine serum (FBS, Gibco) and 100 U/mL penicillin-streptomycin (P/S, Gibco). Cells were seeded in 24-well plates at 3 × 10^5^ cells/mL for the following treatment by nutrients with or without induction to M1 or M2 phenotypes by supplementing 100 ng/mL LPS and 20 ng/mL IFN-γ for 24 h or IL-4 to a final concentration of 20 ng/mL, respectively (31). Upon culturing or treatment with nutrients, all cells were incubated in a humidified incubator at 37°C with 5% CO_2_ supplementation. Adherent cells were gently blown down with 5 mL of serum-free medium, collected by centrifugation at 300 × g for 5 min, and subjected to the following investigation.

### 2.3 Cell viability

Cell viability upon the incubation with EGCG, ginseng extract, or PDX was determined by utilizing CCK-8 solution to generally provide the value concerning the intervention concentration of the complexed nutrients (32). Generally, the RAW264.7 cells were cultured in a 96-well plate at (1 × 10^5^ cells/mL) for 24 h and then incubated with different concentrations (0, 1, 5, 10, 20, 40, 60, 80, 100 μg/mL) of EGCG, ginseng extract, or PDX for 24 h. A blank control group was set up as the wells without cells. After washing with sterile PBS, fresh DMEM medium containing 10 μL of CCK-8 solution was added and incubated for another 2 h. The absorbance was measured at 450 nm (the data were read using SparkControl Magellan 3.1) by using a microplate reader (TECAN Infinite M200 Pro NanoQuant, Switzerland) to evaluate the survival rates of the RAW264.7 cells.

### 2.4 Cell apoptosis and cell cycle analysis

The intervention effect of the nutrient complex on cell apoptosis or cell cycles was determined by flow cytometry. In general, RAW264.7 cells were cultured in a 24-well plate at (3 × 10^5^ cells/mL) for 24 h and then incubated with different concentrations of nutrient complex [ERD-I-H: (E: 2.2 μg/mL, R: 3.9 μg/mL, D: 100 μg/mL); ERD-I-M: (E: 1.1 μg/mL, R: 1.9 μg/mL, D: 50 μg/mL); ERD-I-L: (E: 0.22 μg/mL, R: 0.39 μg/mL, D: 10 μg/mL); ERD-II-H: (E: 7.5 μg/mL, R: 75 μg/mL, D: 100 μg/mL); ERD-II-M: (E: 3.8 μg/mL, R: 37.6 μg/mL, D: 50 μg/mL); and ERD-II-L: (E: 0.75 μg/mL, R: 7.5 μg/mL, D: 10 μg/mL)] for 24 h. A blank control group was set up as the well without nutrient complex supplementation. Cells were then scrapped and collected for the staining with annexin V and propidium iodide (PI) for cell apoptosis analysis. Cell cycle analysis was accomplished by following the well-established protocol (33). Cells were firstly fixed with 75% ethanol and then stained with PI with the presence of RNaseA (20 μg/mL) in 60 min. Cells were then analyzed with BD FACSDiva Software.

### 2.5 Cytokine analysis

The concentrations of TNF-α and IL-1β in the cell culture supernatant were determined with commercial ELISA kits by following the guidelines provided by manufacturer. The standard curve was determined for each test. The absorbance value at 450 nm (the data were read using SparkControl Magellan 3.1) was measured with a microplate reader (TECAN Infinite M200 Pro NanoQuant, Switzerland).

### 2.6 Flow cytometry analysis

RAW264.7 cells were collected after centrifugation at 300 × g for 5 min. Then, the cells were resuspended in 100 μL of cold PBS and pre-incubated with anti-mouse CD16/32 antibody on ice for 10 min to block and prevent non-specific binding with Fc receptors. The cells were washed with PBS by centrifuging at 300 × g for 5 min at 4°C and incubated with APC-conjugated anti-mouse F4/80 and FITC-conjugated anti-mouse CD86 at 4°C for 30 min for staining of M1 macrophages with vortex every 15 min. Excess antibody was removed by washing the cells with cold PBS. The cells were permeabilized in fixation/permeabilization solution (BD Bioscience) for 20 min at 4°C and rinsed in BD perm/wash buffer (BD Bioscience) by centrifuging at 400 × g for 5 min at 4°C. Then, the cells were incubated with PE-conjugated anti-mouse CD206 at 4°C for 30 min to effectively label cells of M2 phenotypes. Excess antibody was removed by washing the cells with cold PBS, and the cells were collected and suspended in 500 μL PBS. Flow cytometric analysis was performed with a BD Symphony A1 (BD, New Jersey, United States) to acquire 10,000 cells in each group for analysis. The data were analyzed with FlowJo software (34).

### 2.7 Macrophage phagocytosis

The phagocytic ability of RAW264.7 macrophages after treating with nutrient complex was determined by the neutral red assay (35, 36). Briefly, the cells seeded in 96-well plates (1 × 10^5^ cells/mL) were stimulated into M1 phenotype and treated with different doses of the nutrient complex (ERD-I-H: (E: 2.2 μg/mL, R: 3.9 μg/mL, D: 100 μg/mL); ERD-I-M: [E: 1.1 μg/mL, R: 1.9 μg/mL, D: 50 μg/mL); ERD-I-L: (E: 0.22 μg/mL, R: 0.39 μg/mL, D: 10 μg/mL); ERD-II-H: (E: 7.5 μg/mL, R: 75 μg/mL, D: 100 μg/mL); ERD-II-M: (E: 3.8 μg/mL, R: 37.6 μg/mL, D: 50 μg/mL); and ERD-II-L: (E: 0.75 μg/mL, R: 7.5 μg/mL, D: 10 μg/mL)] for 24 h simultaneously. The control group was set up as the cells without treating with the nutrient complex. The baseline was determined by setting up the well without cultured cells. After 2 h-incubation with 100 μL of 0.1% neutral red solution, the remaining fraction was removed after washing with ice-cold PBS, and 200 μL of cell lysis buffer (glacial acetic acid: anhydrous acetic acid = 1:1) was added to wells to allow the complete lysis of cells during the following 2 h at room temperature. The absorbance value of the cell lysate was measured at 540 nm (the data were read using SparkControl Magellan 3.1) with a microplate reader (TECAN Infinite M200 Pro NanoQuant, Switzerland).

### 2.8 Statistical analysis

All data were shown as mean ± standard deviation (SD). A two-tailed, unpaired t-test was used to compare the significance of the two groups. One-way ANOVA followed by a Dunnett test (alpha value = 0.05) was used to determine the statistical significance in multiple comparisons. *p*-value ≤0.05 was considered statistically significant and indicated with an asterisk (^*^p < 0.05, ^**^p < 0.01, ^***^p < 0.001, and ^****^p < 0.0001). Statistical analysis was conducted by using GraphPad Prism 7.0 software (GraphPad software, La Jolla, CA, United States).

## 3 Results

### 3.1 Effect of single nutrients on cell viability and inflammation

**Figure 1:**
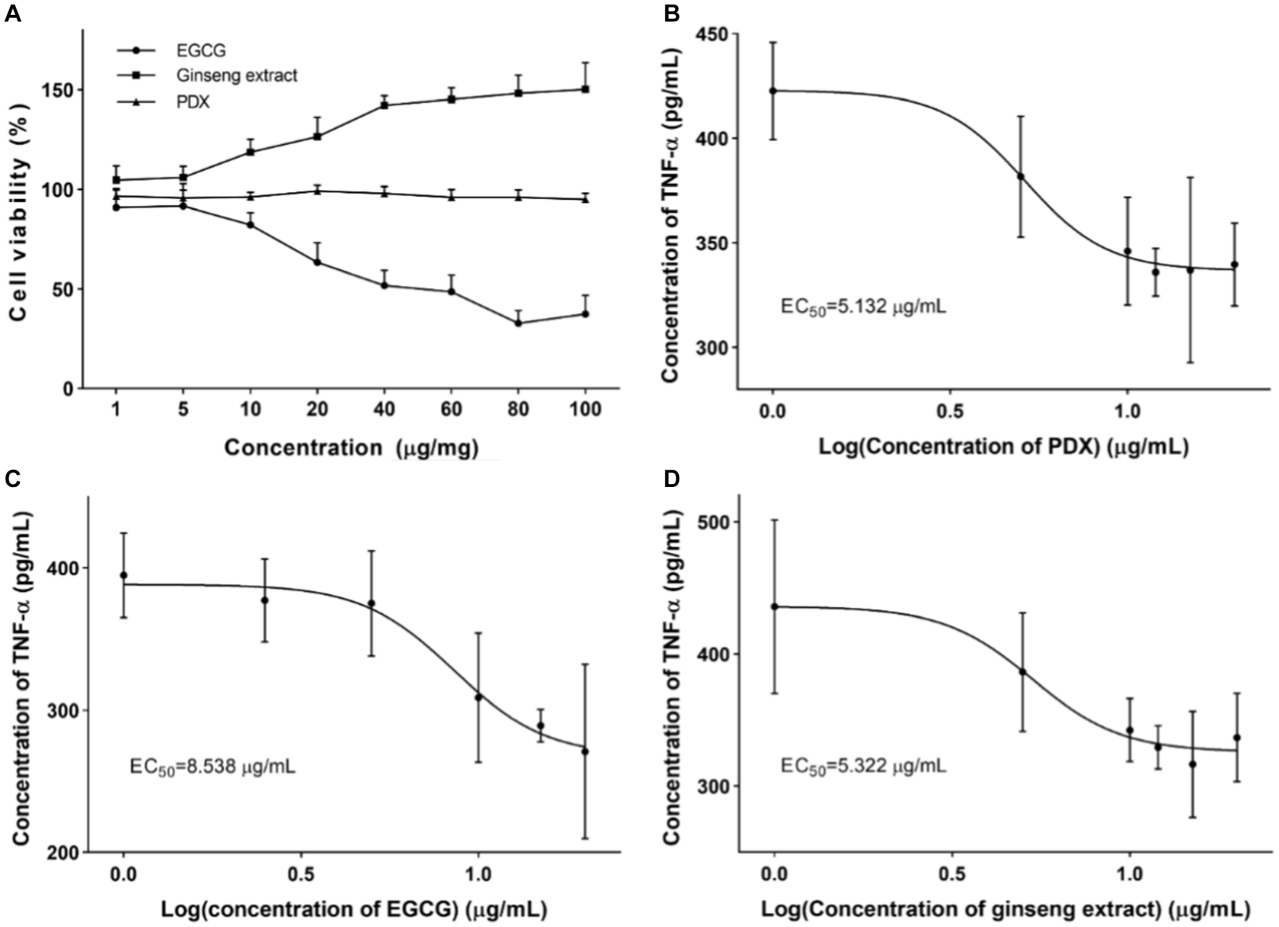
Cell viability and intervention on cell inflammation after treating with corresponding nutrient. **(A)** Effect of EGCG, ginseng extract, and PDX on cell viability was determined after treating RAW264.7 cells with corresponding nutrient to provide data for initial ratio determination. **(B)** Effect of EGCG on the level of TNF-α in cultural supernatant was determined after treating RAW264.7 cells with varied concentration of EGCG. **(C)** Effect of ginseng extract on the level of TNF-α in cultural supernatant was determined after treating RAW264.7 cells with varied concentration of ginseng extract. **(D)** Effect of PDX on the level of TNF-α in cultural supernatant was determined after treating RAW264.7 cells with varied concentration of PDX.

Single nutrients of EGCG, ginseng extract, and PDX have been widely validated as supplements to enhance internal homeostasis or maintain health. In order to verify the intervention effect of the complex of these nutrients, cell viability was assessed as the first step to provide research evidence for the concentration set-up. After treatment of RAW264.7 cells with the corresponding nutrient, cell viability was determined by CCK-8 analysis. As shown in Figure 1A, the result indicated the cytotoxicity of EGCG with a concentration higher than 20 μg/mL. Meanwhile, ginseng extract and PDX were demonstrated to not have a significant effect on cell viability, and ginseng extract was observed to have an effect of promoting cell proliferation in a dose-dependent manner. PDX was verified to not have a significant effect on cell viability even at the concentration of 100 μg/mL. The intervention of different nutrients on cell inflammation was assessed by determining the level of TNF-α after treating LPS-stimulated RAW264.7 with the corresponding nutrient, and the concentration corresponding to half of the effect (EC_50_) was analyzed to provide an initial formula of the complex. The results revealed the dramatic decrease of TNF-α after treating with different nutrients, which indicated the intervention of different nutrients on cell inflammation. EC_50_ was calculated for each nutrient and was used as the initial formulation (Figures 1B–D). The initial nutrient complex was set based on the EC_50_ of individual component as EGCG: ginseng extract: PDX = 1.67: 1.04: 1, which will be used for the initial evaluation of the enhanced effect of the nutrient complex.

### 3.2 Synergistic effect of nutrients

**Figure 2:**
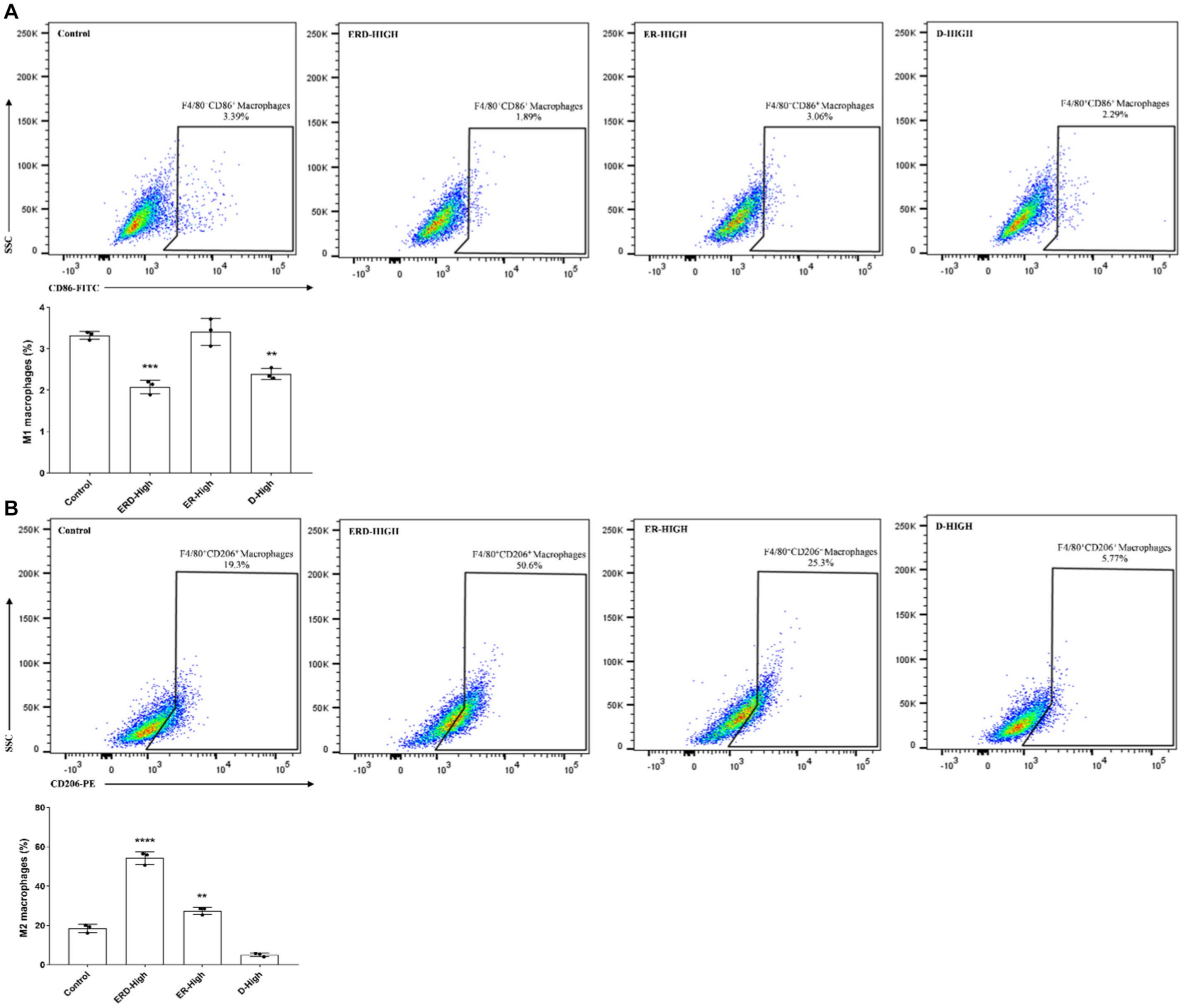
Synergistic effect of nutrients. **(A)** Effect of nutrient complex on M1 macrophage polarization was determined by flow cytometry analysis of RAW264.7 cells after treating with certain concentration of nutrients. The synergistic effect was verified by comparing the groups of the nutrient complex with single or paired nutrient groups to address the effect of PDX. **(B)** Effect of nutrient complex on M2 macrophage polarization was determined by flow cytometry analysis of RAW264.7 cells after treating with certain concentration of nutrients. The synergistic effect was verified by comparing the groups of the nutrient complex with single or paired nutrient groups to address the effect of PDX.

In order to verify the synergistic effect of nutrients or the complex on immunity, the analysis against macrophage polarization was proposed to verify the effect. The intervention on cell polarization was finalized after stimulating RAW264.7 cells with either IFN-γ and LPS or IL-4 to promote M1 or M2 macrophages, respectively. RAW264.7 cells were then cultured and treated with a certain concentration of nutrients or complex (EGCG: 20 μg/mL, ginseng extract: 12.5 μg/mL, PDX: 12 μg/mL) by following the experimental design. Cells were then applied for macrophage polarization analysis after labeling the corresponding cluster of differentiation (CD) with corresponding antibodies to indicate the percentage of M1 (F4/80^+^ CD86^+^) or M2 macrophages (F4/80^+^ CD206^+^). As shown in Figure 2A, the percentage of macrophages with M1 phenotypes was dramatically decreased after treating with nutrient complex, and the enhanced inhibitory effect of the complexed nutrients was demonstrated by comparing with the groups of ER or D, which emphasized the contribution of PDX. The percentage of M2 macrophage was significantly raised (Figure 2B), which indicated the promotion of nutrient complex on M2 macrophages and laterally revealed the inhibition of the nutrient complex on inflammation. Meanwhile, the enhanced effect on M2 macrophage polarization was observed by comparing the results from the nutrient complex with that of single or double nutrients as well. In general, the synergistic effect of nutrients was summarized by analyzing the intervention effect of the nutrient complex on macrophage polarization.

### 3.3 Effect of the nutrient complex on cell apoptosis and cycle

**Figure 3:**
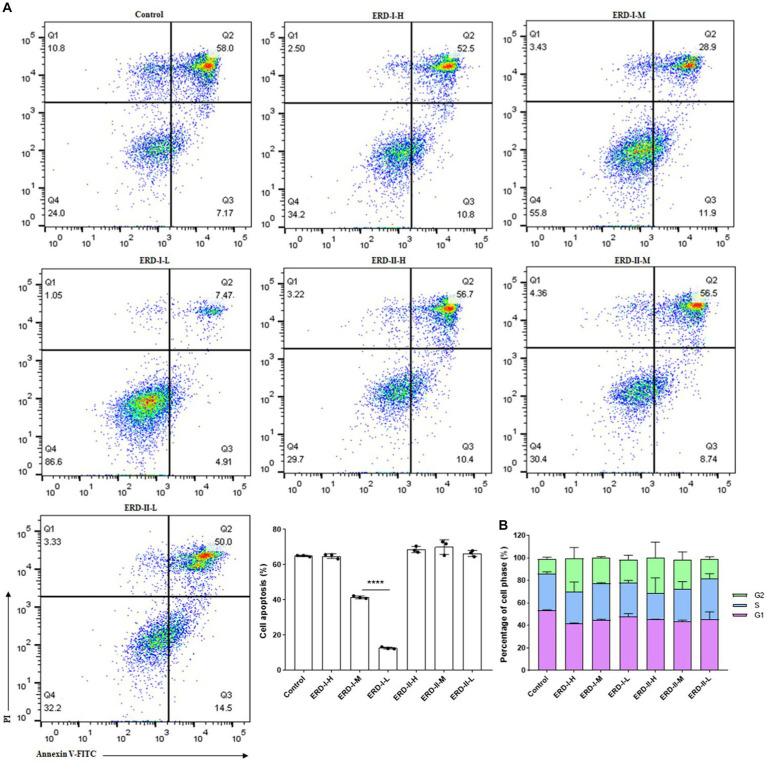
Effect of the nutrient complex on cell apoptosis and cycle. **(A)** Effect of nutrient formulas on cell apoptosis was determined by treating RAW264.7 cells with certain concentrations of formulas I and II. The significant inhibition of nutrient formulas on cell apoptosis could be summarized for formula I in a dose-dependent manner, whereas no significant effect of formula II was observed. **(B)** Effect of nutrient formulas on cell cycles was determined by treating RAW264.7 cells with certain concentrations of formulas I and II. The significant promotion of nutrient formulas on cell cycles to the G2 phase could be summarized for formulas I and II in a dose-dependent manner in general.

Based on the results concerning the synergistic effect of nutrients, two alternative formulas of the nutrient complex with the ratio of EGCG: ginseng extract: PDX = 1: 1.79: 46.16 (formula I coined ERD I) and 1: 10: 13.3 (formula II coined ERD II) were set up to highlight the effect of PDX in ERD I and the effect of both ginseng extract and PDX in ERD II. Then, the intervention effect of these nutrient complexes on cell growth or proliferation was analyzed. The concentration was set up to avoid the cytotoxicity of EGCG and classified as the concentration of PDX: high for 100 μg/mL, medium for 50 μg/mL, and low for 10 μg/mL. The concentration of EGCG and ginseng extract was calculated from the value of PDX based on the defined corresponding formula. Cell apoptosis was analyzed by following the steps described above, and the effect on cell cycles was determined by performing flow cytometry. The result indicated that formula I inhibited the cell apoptosis dramatically, whereas no significant effect of formula II was observed (Figure 3A), which indicated the significant contribution of PDX on cell promotion, and the increased concentration of ginseng extract was considered to contribute negatively. Cell cycle analysis revealed the promotion of both formulas on cells in the G2 phase, which unveiled the promotion of both formulas on cell proliferation (Figure 3B) to maintain the cells in the phase of reproduction, thus potentially contributing to self-renewal capacity.

### 3.4 Effect of the nutrient complex on inflammation-related cytokine

**Figure 4:**
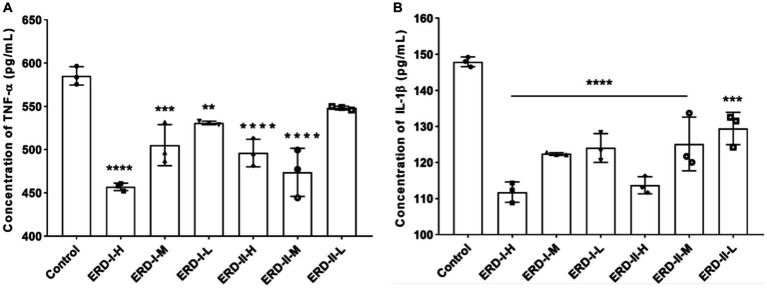
Effect of nutrient formulas on secretion of inflammation-related cytokines. **(A)** Effect of nutrient formulas on the level of TNF-α in cultural supernatant was determined after treating RAW264.7 cells with certain concentrations of formulas I and II. **(B)** Effect of nutrient formulas on the level of IL-1β in cultural supernatant was determined after treating RAW264.7 cells with certain concentrations of formulas I and II.

The effect of formulas I and II on cell inflammation was reflected by determining the level of inflammation-related cytokines, namely, TNF-α and IL-1β, to indicate the inflammation status of cells. RAW264.7 cells were firstly stimulated with LPS and IFN-γ to establish an inflammatory model and then treated with different concentrations of the nutrient complex. The level of corresponding cytokines was determined by ELISA with commercial kits, and the results revealed a significant inhibition on the level of TNF-α (Figure 4A) and IL-1β (Figure 4B) upon treatment with a corresponding nutrient formula. The intervention effect of the nutrient complex on the corresponding cytokines was determined in a dose-dependent manner in general. The results revealed no significant variation of the two formulas on the level of cytokines, which demonstrated the intervention of the formulas on cell inflammation.

### 3.5 Effect of the nutrient complex on macrophage polarization

**Figure 5:**
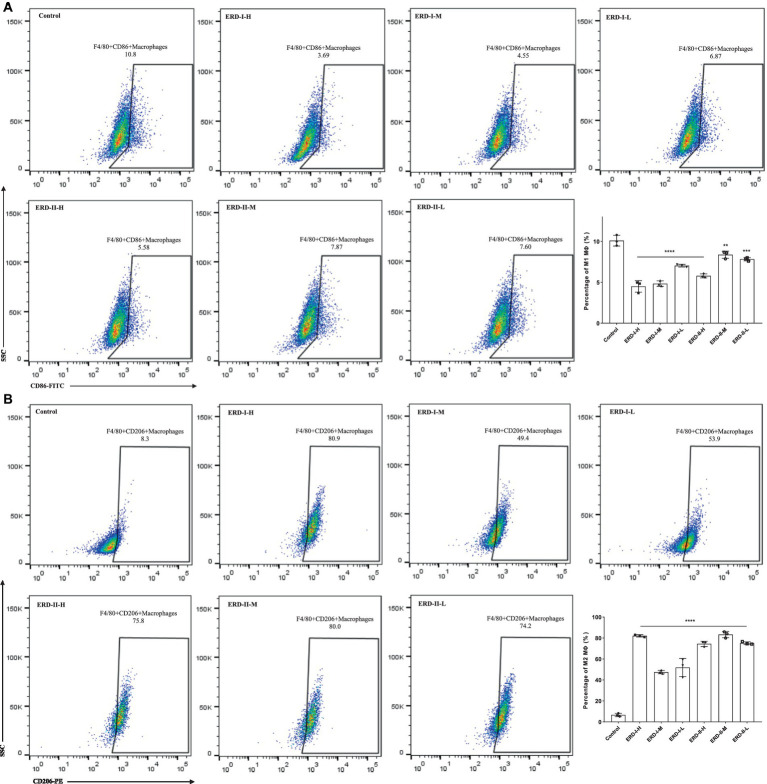
Effect of nutrient formula on macrophage polarization. **(A)** Effect of nutrient formulas on the macrophage polarization to M1 phenotype was determined by flow cytometry analysis of RAW264.7 cells after treating with certain concentrations of nutrient formulas I and II. **(B)** Effect of nutrient formula on the macrophage polarization to M2 phenotype was determined by flow cytometry analysis of RAW264.7 cells after treating with certain concentrations of nutrient formulas I and II.

The effect of the nutrient complex on macrophage polarization was analyzed with flow cytometry by following the steps described above. In general, cells were stimulated to the M1 phenotype macrophage after incubation with IFN-γ and LPS, with M2 macrophage stimulated with IL-4. Then, cells were treated with different concentrations of formulas I and II. The percentage of M1 and M2 macrophages were quantified, and the results revealed the significant inhibition of both formulas on M1 polarization, which indicated the intervention of the nutrient complex on inflammation-related macrophages (Figure 5A). Meanwhile, both formulas could promote the polarization of macrophages to the M2 phenotype to exhibit an anti-inflammation effect (Figure 5B).

### 3.6 Effect of the nutrient complex on macrophage phagocytosis

**Figure 6:**
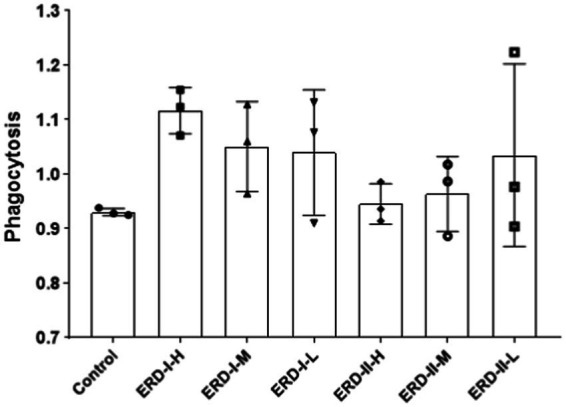
Effect of nutrient formulas on macrophage phagocytosis. Cells were treated with certain concentrations of nutrient formulas of I and II and then analyzed by neutral red assay.

Phagocytotic capacity represents the ability of macrophages in innate immune defense. In order to verify the intervention effect of the nutrient complex on macrophage-mediated immune defense, cell phagocytosis was analyzed by using neutral red assay. After the treatment of cells with different formulas of the nutrient complex, cells were applied for neutral red assay. The phagocytotic capacity was determined after recording the absorbance value. As shown in Figure 6, the results indicated the intervention of both formulas on phagocytosis to reveal the increased phagocytosis after treatment with both formulas, though no significance was observed. Meanwhile, the enhanced effect of formula I was observed by comparing with the control or formula II groups, which suggested the better intervention of formula I to address the functionality of PDX rather than the increased concentration of ginseng extract.

## 4 Discussion

The daily intake of varied types of food nutrients is recommended to fulfill the requirement of health maintenance (37). This general investigation has explored the intervention of single nutrients on health and disease from distinct aspects to validate their bioactivities or provide recommended ingestion (38, 39). However, the complex formula including varied types of nutrients has not been reported thoroughly to indicate its comprehensive effect or synergistic contribution on certain healthy states. Thus, we have proposed to evaluate the complex effect of the nutrients EGCG, ginseng extract, and PDX as the representatives of polyphenol, flavone or polysaccharides, and dietary fiber to generally cover the daily food intake. The comprehensive effect has been evaluated by determining the bioactivities of the complex formula on cell viability, inflammation, or macrophage polarization. The results are expected to provide data to inspire following investigations on complex formulas or indicate the supplementation of corresponding nutrients in foods.

The distinct bioactivities of selected nutrients were observed by determining cell viability after treating with corresponding nutrients individually to indicate the maintained or promoted effect of PDX and ginseng extract, respectively. Meanwhile, the significant effect on cell viability was visualized upon exposure to a high concentration of EGCG. Thus, the recommended dosage for each nutrient was determined based on the effect of EGCG to form the initial formula of the nutrients for baseline investigation on cell inflammation or functionalization. The results indicated the synergistic effect of the fixed ratio on certain cell activities of macrophage polarization to M1 or M2 phenotypes, which reflected the enhanced efficiency of the formula on the promoted effect on M2 polarization to indicate the anti-inflammatory effect. Thus, it is evident that the complexed nutrient could potentially enhance the intervention effect.

Moreover, the nutrient complex with different formulas could possibly exhibit varied intervention effects. Thus, the alternative ratio of selected nutrients has been designed to emphasize the contents of either PDX (ERD I) or ginseng extract (ERD II) to exhaustively explore the intervention of the defined formula. The results revealed the distinctive effect of the different formulas on cell apoptosis or cycles and the effect on macrophage polarization. It was revealed that ERD I showed a dramatically inhibitory effect on inflammation, cell apoptosis and M1 polarization, which demonstrated an enhanced effect upon ingestion of raised level of PDX by comparing with the formula with a decreased ratio of PDX and increased concentration of ginseng extract. The effect of different formulas on the expression level of the inflammation-related cytokines TNF-α and IL-1β has verified the reinforced impact of ERD I. Thus, it was demonstrated that the complex formula with enhanced ingestion of PDX is expected to maximize the beneficial effect of the complex formula to potentially indicate the supplementation in food manufactures. The data are expected to inspire future research and application to focus on not only the selected nutrients in complex formulas but also the ratio of each nutrient.

## 5 Conclusion

Due to the necessity to explore the comprehensive effects of nutritional formulas on either health or disease, the representative prebiotics of EGCG, ginseng extract, and PDX were selected on behalf of the classification of polyphenol, flavone or polysaccharides, and dietary fiber in this study to generally cover the daily food intake to reveal the intervention effects on cell inflammation and macrophage polarization *in vitro*. It was demonstrated that the nutrient complex produced a synergistic effect on certain cell activities of inhibition on M1 macrophage polarization and promotion on M2 macrophage polarization. The intervention effect was then verified by treating cells with two fixed formulas, which revealed the inhibition on M1 macrophage polarization and M1-mediated inflammation and promotion on M2 macrophages for anti-inflammatory effect, as well as a generally enhanced effect on cell phagocytosis. In general, the formula containing EGCG, ginseng extract, and PDX was demonstrated to possess an enhanced immunomodulatory effect on cell inflammation and macrophage polarization. The results are expected to provide research evidence for future supplementation in food with prescribed functionality or inspire further research on the effect of nutritional formulas on health and diseases.

## Data availability statement

The original contributions presented in the study are included in the article/supplementary material, further inquiries can be directed to the corresponding author.

## Author contributions

YW: Data curation, Formal analysis, Methodology, Software, Writing – original draft, Writing – review & editing. YH: Conceptualization, Data curation, Funding acquisition, Investigation, Methodology, Software, Visualization, Writing – original draft, Writing – review & editing. ZN: Resources, Writing – review & editing. XZ: Methodology, Writing – review & editing. DF: Investigation, Writing – review & editing. XJ: Methodology, Writing – review & editing. HL: Methodology, Writing – review & editing. SW: Conceptualization, Funding acquisition, Investigation, Project administration, Supervision, Writing – review & editing. YZ: Resources, Writing – review & editing.
